# Reducing Radiation Dermatitis for PBS Proton Therapy Breast Cancer Patients Using SpotDelete

**DOI:** 10.1016/j.ijpt.2024.100628

**Published:** 2024-08-28

**Authors:** Samantha G. Hedrick, Laura Buchanan, Stephen Mahan, Chester Ramsey

**Affiliations:** Thompson Proton Center, Knoxville, TN 37909, USA

**Keywords:** PBS, Breast, Dermatitis, Side effects

## Abstract

**Purpose:**

The purpose of this work was to reduce the severity of radiation dermatitis for breast cancer patients receiving pencil beam scanning proton therapy. The hypothesis was that eliminating proton spots (SpotDelete) in the 0.5 cm skin rind would reduce the potentially higher relative biological effectiveness (RBE) known to occur at the Bragg Peak.

**Patients and Methods:**

Our center has been using an in-house developed Python script in RayStation since 2021 to remove spots from the skin rind of breast patients. In this work, we retrospectively reviewed the on-treatment visit data from a cohort of breast patients treated with hypofractionation (16 fractions) before this technique (MinDepth) and after (SpotDelete) to acquire the physician-reported radiation dermatitis scores. We evaluated the delivered treatment plans, calculating the linear energy transfer (LET) and applying 3 variable RBE models, Carabe-Fernandez, Wedenberg, and McNamara. An α/β of 10 was assumed for the skin.

**Results:**

In the MinDepth cohort (*n* = 28), grade 1, 2, and 3 dermatitis accounted for 57%, 36%, and 7% of the cases, respectively. For SpotDelete (*n* = 27), the incidence rate of grade 1 and 2 acute radiation dermatitis was 67% and 37%, respectively. There were 0 instances of grade 3 dermatitis observed in the SpotDelete cohort. The onset of radiation dermatitis in the SpotDelete cohort was delayed compared to MinDepth, occurring 1 week later in the course of treatment. There was no significant difference in LET or in any of the variable RBE models when analyzing the 0.5 cm skin rind between the cohorts.

**Conclusion:**

Despite the lack of correlation in LET or RBE, SpotDelete has been shown to reduce the severity and onset of radiation dermatitis. Possibly, more research into the α/β for skin and RBE models based on skin cell lines could provide insight into the efficacy of the SpotDelete technique.

## Introduction

Proton therapy for breast cancer can provide improved heart and lung sparing compared to photon therapy.^1^ Traditionally, using passive or double-scattering methods, the skin dose was significantly higher in proton therapy compared to photon therapy due to the nature of the spread-out Bragg peak (SOBP). Because the SOBP had to deliver the dose to the deepest portion of the target, along the apex of the breast for an *en face* field, this led to a dose as high as prescription at the skin, particularly along the medial and lateral portions of the breast. The development of pencil beam scanning (PBS) proton therapy allowed for improved skin sparing because the SOBP could now be customized along each beam path, that is, the skin sparing at any point on the breast was now only dependent on the depth of the target along that point in the beam. However, published studies still report higher radiation dermatitis scores in PBS proton therapy compared to photon therapy.^2^ Multiple clinical trials have documented the maximum rates of acute radiation dermatitis in breast cancer patients receiving proton therapy.^2^, ^3^, ^4^, ^5^, ^6^, ^7^, ^8^ These studies revealed that between 70.7% and 100% of patients experienced at least grade 2 acute radiation dermatitis, characterized by moderate to brisk erythema, moderate edema, or moist desquamation. Additionally, grade 3 acute radiation dermatitis, involving moist desquamation or bleeding, was observed in 5.1% to 43% of breast patients treated with protons. Several factors can impact the risk of severe radiation dermatitis, including factors related to the treatment delivery and the patient's individual characteristics. The degree of injury is directly proportional to the radiation dose on the skin.^3^ The skin's sensitivity to radiation varies across different regions. The anterior neck, limbs, chest, abdomen, and face are among the most sensitive areas.^9^ In addition, breast tissue shows higher radiosensitivity compared to other body regions. Patient-related factors include obesity, age, gender, prolonged sun exposure, and smoking habits.^9^, ^10^ Furthermore, older individuals and females are more likely to experience radiation skin damage.^9^, ^10^ The purpose of this study is to evaluate treatment planning methods in PBS proton therapy to reduce radiation dermatitis and provide patients skin sparing that is as good, if not better, than photon results.

The relative biological effectiveness (RBE) of proton therapy is typically a constant value of 1.1 in treatment planning systems.^11^, ^12^ Thus, it is considered that protons are 10% more biologically effective than photons. However, it is well known that the RBE varies along the length of the Bragg peak, <1.1 in the entrance region and >1.1 at the distal end. The greater RBE at the distal end of the Bragg peak is due to higher dose-weighted linear energy transfer (LETd).^13^, ^14^, ^15^ As the Bragg peak rises rapidly in dose, so does the LETd. The RBE is dependent on linear energy transfer (LET), but it is also dependent on the α/β ratio of the tissue in the beam path. Because the RBE is dependent on multiple factors, most treatment planning systems do not currently account for variable RBE. Treatment planners typically consider this phenomenon in their decision-making process, choosing not to end treatment beams on critical structures, on the understanding that the RBE is higher than visualized, but it is unknown exactly how much higher.^14^ This is to mitigate the so-called “end-of-range” effect. In breast proton therapy, researchers have evaluated the impact of the end-of-range effect using *en face* beams on rib fractures.^16^ When considering skin dose for these *en face* fields, most may not consider this end-of-range effect because the skin is in the entrance region of the beam rather than at the point at which they stop. However, the same concept still applies. In PBS, the beam is made up of multiple energy layers, thus depositing Bragg peaks, or “spots,” throughout the entirety of the target.^17^ For each Bragg peak, there is a higher LET at the point of the spot than in the entrance region. Ideally, high LET would be focused on the target and out of organs-at-risk (OAR). In the case of the breast, typically, a 0.5 cm skin rind is excluded from the target and is then considered an OAR. We hypothesized that removing spots from the 0.5 cm skin rind would reduce dermatitis by eliminating higher LET out of the skin.

## Patients and Methods

In 2021, we developed a script to remove spots out of any region of interest chosen by the user, a method we call SpotDelete.^18^ Some treatment planning systems allow users to choose a minimum depth at which spots can be placed, effectively preventing spots in the skin for an *en face* field. However, this method does not work for beams that intersect the skin tangentially or end on the skin. Our script can remove skin spots, regardless of the beam path. We have previously proved that deleting skin spots out of the skin rind did not negatively affect the nominal dose distribution or the robustness. Because the plans met physician criteria and were considered safe and deliverable, we began treating every breast and chest wall patient using the SpotDelete method, deleting spots out of the 0.5 cm and 0.3 cm skin rind, respectively.

To evaluate the efficacy of this scripted method in reducing radiation dermatitis, we performed a retrospective study of patients who underwent PBS proton therapy at our center between January 2019 and February 2023, with a cohort of patients treated before using SpotDelete (referred to as MinDepth) and with SpotDelete. The patients treated before SpotDelete were planned using the minimum radiological depth tool, which prevented spots in the first 0.5 cm of the beam path. Those treated with SpotDelete had spots removed from the entire 0.5 cm skin rind. Patients with stage IV breast cancer diagnosis were excluded from this study. This retrospective study involved analyzing the medical records and treatment data for all breast patients treated with hypofractionated proton therapy. Patients were treated with PBS, and treatment plans were created using 1 to 2 *en face* beams, between 0 and 60 degrees, to deliver hypofractionated breast treatment in 16 fractions, optionally followed by a 4-fraction boost. Patients were simulated on a breast board, and plans were developed on the 3D Computed Tomography (CT) scan without deep inspiration breath-hold. All patient plans were developed using RayStation v8a (RaySearch Laboratories, Stockholm, Sweden). A water-equivalent 7.5-cm Lexan ranger shifter was used for each beam, attempting to reduce the air gap to the breast to close as possible. The plan was robustly optimized with a setup uncertainty of 0.5 cm and a 3.5% range uncertainty. The Monte Carlo algorithm was used for optimization, and the final dose was computed using Monte Carlo with 0.5% statistical uncertainty. During weekly on-treatment visits (OTVs), the patients were evaluated for radiation dermatitis by physicians and nursing team members using the Common Terminology Criteria for Adverse Events (CTCAE v4) scale.^19^ This grading system scored dermatitis from grade 1 (faint erythema/dry desquamation) to grade 3 (moist desquamation). This weekly OTV data were collected for each patient. Data were not collected after the last day of treatment. Additionally, the dose statistics for the Breast_Clinical Target Volume (CTV), as defined by RTOG,^20^ were collected, including the dose to 95% of the volume (D95%), the volume receiving prescription (VRx), and the maximum dose in the CTV (Dmax). The skin OAR (Skin5mm) was defined as the 0.5 cm skin rind within 1 cm of the Breast_CTV. We evaluated the maximum dose, dose to 1cc (D1cc), dose to 2cc (D2cc), dose to 5cc (D5cc), volume receiving prescription, and volume receiving 90% of the prescription dose (V90). Each of the listed Skin5mm dose statistics was evaluated on the nominal dose, with a constant RBE = 1.1. The LET was evaluated using the RayStation calculation. RayStation computed dose-averaged LET in water normalized to the density of each voxel, accounting for tissue heterogeneity. Linear energy transfer from primary and secondary protons is included, but LET from heavier fragments is not. The volume of Skin5mm receiving 2 KeV/µm (V2 keV/µm), V3 keV/µm, and average LET was analyzed. Additionally, to evaluate the impact of LETd, we calculated the variable RBE dose using 3 LET-weighted RBE models: Carabe-Fernandez,^21^ Wedenberg,^22^ and McNamara.^23^ For each variable RBE model, we chose to keep a constant α/β ratio equal to 10. There is very little consensus on the α/β of skin, ranging from 2.5 to 10.^24^, ^25^ The dose statistics for the Skin5mm were evaluated on each of the 3 variable RBE models and are reported. Statistical analyses were performed using a Student *t* test.

## Results

We evaluated 28 patients planned with MinDepth and 27 patients planned with SpotDelete. Patient characteristics are shown in Table 1. No significant difference was noted in patient age or breast CTV volume between the 2 cohorts. On-treatment visit data were acquired only while patients were under beam, and the median follow-up time was at fraction 18 for both the MinDepth and SpotDelete cohorts.

**Table 1:** Patient characteristics.

	MinDepth	SpotDelete	*P*
Total patients	28	27	
Average age (years)	65	63	.41
Target			
Right breast	6	5	
Left breast	22	22	
Average CTV volume (cm^3^)	943	852	.34
Dose in 16 fractions			
4240 Gy_RBE_	5	0	
4256 Gy_RBE_	15	18	
4272 Gy_RBE_	8	9	
Boost			
10 Gy_RBE_ in 4 fractions	26	25	
None	2	2	
Number of beams			
1	10	0	
2	18	27	
Planning type			
SFO	23	2	
Hybrid	0	25	
MFO	5	0	

Abbreviations: SFO, single-field optimization; MFO, multiple-field optimization.

For each of the clinically delivered plans in both patient groups, the breast CTV received at least D95% > 95% of the prescription in the initial phase of treatment. Most patients, 26/28 for MinDepth and 25/27 for SpotDelete, received an additional 10 Gy_RBE_ boost in 4 fractions. In these results, we report the dose from the initial phase only. The maximum dose in the breast was, on average, 105.7% of prescription for MinDepth and 106.3% of prescription for SpotDelete. All plans in both cohorts were planned using our in-house robust evaluation criteria of CTV D97% > 97% on robust perturbations.

Based on our standard planning technique in 2019-2020, most (23/28) of the MinDepth patients were planned using a single-field optimization (SFO) technique. For those patients with 2 beams, an SFO plan used an equal contribution of dose from both beams with a uniform dose distribution per beam. The maximum dose per beam was no more than 52% of the prescription dose. The 5 remaining patients in the MinDepth cohort were planned using a multiple-field optimization (MFO) technique. This was not intentional and was found during this retrospective review. Our planning technique evolved in 2021, and we began to use a technique we call “120MFO.” Rather than requiring a uniform dose distribution from each of the 2 treatment beams, a small gradient is introduced. Each of the 2 treatment beams can deliver no less than 40% of the prescription dose and no more than 60% of the prescription dose. This allows better target conformality and OAR sparing without sacrificing the inherent robustness of an SFO technique. Most (25/27) of the SpotDelete cohort patients are planned with this 120MFO technique in conjunction with the SpotDelete technique.

In the MinDepth cohort, grade 1 dermatitis accounted for 57% of cases, grade 2 for 36%, and grade 3 for 7%. In the SpotDelete cohort, the incidence rate of grade 1 acute radiation dermatitis was 67%, and the incidence of grade 2 acute radiation dermatitis group was 37%. There were 0 instances of grade 3 dermatitis observed in the SpotDelete cohort, as shown in Table 2. For the 5 patients accidentally planned using MFO in the MinDepth cohort, 2 experienced grade 1 dermatitis, and 3 experienced grade 2. There does not appear to be an impact of the SFO versus MFO planning technique for this small subset of patients.

**Table 2:** Dermatitis rates for MinDepth and SpotDelete cohort compared to published studies.

Maximum dermatitis severity (CTCAE v4)	Proton PBS MinDepth	Proton PBS SpotDelete	Photon Shaitelman 2015	Photon Schmeel 2020
	*n* = 28	*n* = 27	*n* = 138	*n* = 140
Grade 0	0%	0%	6%	21%
Grade 1	57%	67%	58%	51%
Grade 2	36%	37%	36%	27%
Grade 3	7%	0%	0%	0%

Abbreviations: CTCAE v4, Common Terminology Criteria for Adverse Events; PBS, pencil beam scanning.

In the MinDepth cohort, the 2 patients without a sequential boost developed grade 1 radiation dermatitis, both during week 3 of treatment. In the SpotDelete cohort, of the 2 patients who did not receive a sequential boost, 1 patient developed grade 1 radiation dermatitis, reported in week 3, and 1 developed grade 2 radiation dermatitis, reported in week 4 of treatment.

The onset of radiation dermatitis in the SpotDelete cohort was delayed compared to the MinDepth cohort, occurring at a median value of fraction 11, during week 3 of treatment, for SpotDelete and fraction 9, during week 2 of treatment, for MinDepth. Similar to previous studies, we found that larger breast volume was associated with a higher dermatitis score.^26^ For patients with grade 0 and 1 radiation dermatitis, grouped from both cohorts, the average CTV volume was 817 cm^3^, and for those with grade 2 and 3 radiation dermatitis, the average CTV volume was 1030 cm^3^ (*P* = .04).

**Figure 1:**
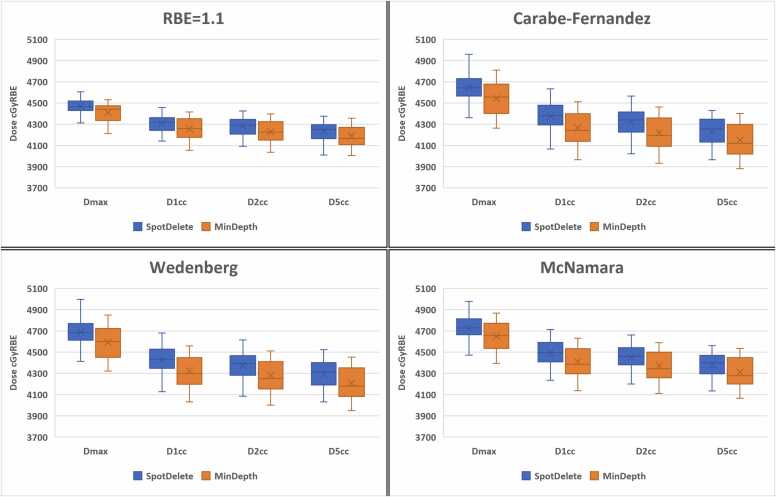
Box and whisker plots for each of the RBE dose evaluations, reporting the Skin5mm dose statistics for the MinDepth (orange) and SpotDelete (blue) cohorts. Abbreviation: RBE, relative biological effectiveness.

**Figure 2:**
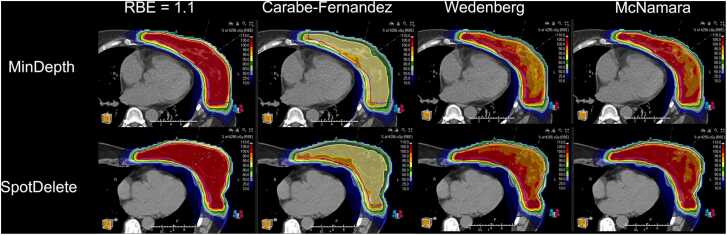
Dosimetric comparison for MinDepth and SpotDelete cohorts, including nominal RBE = 1.1 and 3 variable RBE models. Abbreviation: RBE, relative biological effectiveness.

There was no significant difference in LET between the 2 cohorts, as shown in Table 3, when evaluating the V2 keV/µm, V3 keV/µm, or average LET. In all evaluations of RBE, including the constant RBE = 1.1 and the 3 variable RBE models, the SpotDelete cohort skin dose was higher than the MinDepth cohort for all dose metrics, as shown in Figure 1. The statistical analysis indicated a significant difference (*P* < .05) between the 2 cohorts for each metric and each model; however, the clinical difference is small. The volumetric data, V90% and V100%, were similarly higher for SpotDelete, but there was no significant difference between the cohorts. The table of data for dose and volume is included in the Supplemental Material. Figure 2 demonstrates the 4 RBE models for a MinDepth patient (top) and SpotDelete patient (bottom).

**Table 3:** LET evaluation for MinDepth and SpotDelete cohorts.

Skin LET	MinDepth	SpotDelete	*P*
V2 keV/µm (cm^3^)	32.26	35.12	.35
V3 keV/µm (cm^3^)	1.86	3.79	.12
Average (keV/µm)	0.04	0.61	.69

Abbreviation: LET, linear energy transfer.

## Discussion

The dermatitis incidence rates in the MinDepth cohort align with findings from previously published studies for patients undergoing proton therapy.^2^, ^3^, ^4^, ^5^, ^6^, ^7^, ^8^ This indicates the consistency of our experience with other proton centers. The dermatitis incidence rates in the SpotDelete cohort align with findings from previous photon-based randomized trials.^27^, ^28^ In those trials, rates of grade 1 dermatitis ranged from 51% to 58%, while rates of grade 2 dermatitis ranged from 27% to 36% when using hypofractionation techniques. These published photon studies provided a benchmark for our SpotDelete results, and it was encouraging to see the impact of our technique in reducing radiation dermatitis to the level of photon results.

A surprising result from the study was the lack of correlation between the reduced dermatitis with SpotDelete, as demonstrated by the OTV results, and the LET or variable RBE model doses. We anticipated that at least one of the models would show a reduction in dose to the skin rind based on the hypothesis that removing spots will reduce LET. A limitation of this study was the use of a single α/β ratio, particularly considering the lack of consensus on the α/β for skin. Originally, we had run our analysis for a lower α/β = 3.76. The analysis proved similarly uncorrelated between the MinDepth and SpotDelete cohorts. The data presented here are for an α/β = 10 Gy, and the α/β = 3.76 data are available in the Supplemental Material. Additionally, each of the RBE models is applicable in different situations. For example, the University of Florida group describes their choices of RBE models based on the cell lines that informed the model.^29^ To our knowledge, no RBE model has been developed using skin cell lines. Potentially, a skin-informed model could demonstrate the expected dose difference between the MinDepth and SpotDelete techniques.

One of the compounding factors in our study was the transition from a fully SFO planning technique to a 120MFO planning technique. It can be hypothesized that perhaps the planning technique change is the reason for the change in dermatitis results, rather than the SpotDelete technique. However, it seems unlikely. We have done evaluations, under review in another publication, comparing the dosimetric results of SFO versus 120MFO. We found that changing the planning technique did not inherently change the LET or variable RBE results for breast plans. The advantage of the 120MFO technique is the ability to provide slightly better conformality around the lung and heart. Additionally, with less restrictions on per-beam homogeneity, the 120MFO technique better accommodates the use of SpotDelete.

For the nominal RBE = 1.1 dose and each of the variable RBE models, the skin dose was higher for all metrics in the SpotDelete cohort compared to MinDepth. While the differences were statistically significant, they do not appear to be clinically significant. For most of the dose values, the differences are <1 Gy_RBE_. At the onset of using this SpotDelete technique, there were concerns there would be a rind of higher dose, just distal to the skin rind, due to the optimizer attempting to maintain target coverage at the skin rind interface. However, we do not see this effect in the LET or in any of the RBE dose evaluations.

There are other limitations of this study, including the potentially inconsistent reporting for CTCAE values during OTVs. In our clinic, different nurses or physicians may be reporting these values each week. Additionally, there is a small sample size for this study, with fewer than 30 patients in each cohort. Finally, skin reactions can be impacted by several factors, including chemotherapy, body mass index, age, sun exposure, smoking status, and others. These factors were not accounted for in this study. In fact, most of these data are not necessarily collected for the patient. These limitations led to the development of a clinical trial, evaluating this technique for breast and chest wall patients.^30^ In this trial, we will prospectively collect photographs of the patients’ skin before treatment, each week during treatment, and at 1 month and 6 months post treatment, to be evaluated by a single reviewer to report radiation dermatitis. Additionally, the patients will complete a quality of life survey each week during treatment and each week for 5 weeks post treatment.

## Conclusions

Using physician-reported outcomes, we have shown that deleting proton spots out of the 0.5 cm skin rind for hypofractionated PBS treatment of breast cancer can reduce the severity of radiation dermatitis and delay the onset of reactions. Deleting spots is an efficient technique to reduce skin side effects and does not negatively impact target coverage or plan robustness.

## Author Contributions

Samantha Hedrick: Conceptualization, Data curation, Formal analysis, Investigation, Methodology, Validation, Visualization, Writing- Original draft, Writing- Review and Editing. Laura Buchanan: Visualization, Writing- Review and Editing. Stephen Mahan: Writing- Review and Editing. Chester Ramsey: Conceptualization, Data curation, Formal analysis, Investigation, Methodology, Validation, Visualization, Writing- Review and Editing.

## Declaration of Conflicts of Interest

The authors declare that they have no known competing financial interests or personal relationships that could have appeared to influence the work reported in this paper.

## Data Availability

The data that support the findings of this study are available from the corresponding author upon reasonable request.

## Footnotes

Supplementary data associated with this article can be found in the online version at doi:10.1016/j.ijpt.2024.100628.
