# Supplementary material for: Reducing Radiation Dermatitis for PBS Proton Therapy Breast Cancer Patients Using SpotDelete
Source: Int J Part Ther. 2024 Aug 28;13:100628. doi: 10.1016/j.ijpt.2024.100628 (PMC11408802; doi:10.1016/j.ijpt.2024.100628)

Supplemental data:

Skin doses evaluated at an alpha/beta = 3.76 Gy


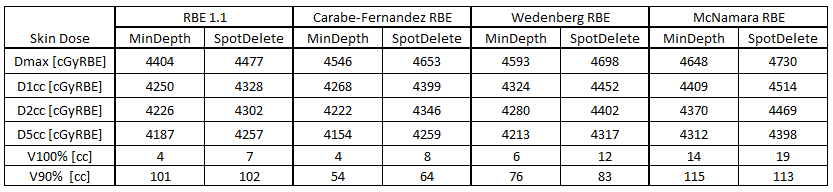


Skin doses evaluated at an alpha/beta = 10 Gy


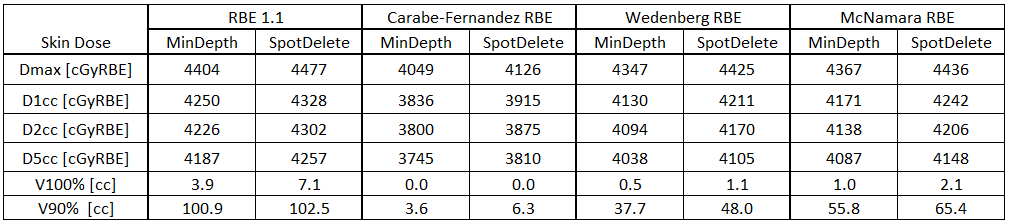

Supplement: Supplementary file 1 — Supplementary material. [file mmc1.docx]
